# Brain Interleukin-17A contributes to neuroinflammation and cardiac dysfunction in rats with myocardial infarction

**DOI:** 10.3389/fnins.2022.1032434

**Published:** 2022-10-13

**Authors:** Yang Yu, Robert M. Weiss, Shun-Guang Wei

**Affiliations:** ^1^Department of Internal Medicine, University of Iowa, Iowa City, IA, United States; ^2^Abboud Cardiovascular Research Center, University of Iowa, Iowa City, IA, United States; ^3^Iowa Neuroscience Institute, University of Iowa, Iowa City, IA, United States; ^4^Iowa City VA Health Care System, Iowa City, IA, United States

**Keywords:** hypothalamic paraventricular nucleus, cytokine, inflammation, sympathetic nervous system, heart failure, interleukin-17RA, chemokine, brain

## Abstract

Proinflammatory cytokines produced outside the central nervous system can act in the brain to promote sympathetic activation that contributes to the progression of heart failure (HF). Interleukin (IL)-17A, a key inflammatory regulator which orchestrates immune responses to promote chronic inflammation, has been implicated in the pathophysiology of HF. We previously reported that IL-17A acts within the brain, particularly in the hypothalamic paraventricular nucleus (PVN), to increase expression of inflammatory mediators and, consequently, sympathetic outflow. The present study sought to determine whether IL-17A levels are elevated in a rat model of HF induced by myocardial infarction and, if so, whether increased expression of IL-17A in the brain itself contributes to neuroinflammation and cardiac dysfunction in this disease setting. Male SD rats underwent coronary artery ligation (CL) to induce HF or sham operation (SHAM). Compared with SHAM rats, HF rats exhibited significantly increased IL-17A levels in plasma, beginning within 1 week with a peak increase at 4 weeks after CL. IL-17A levels in cerebrospinal fluid (CSF) were also increased in HF rats and correlated with IL-17A levels in the plasma. The mRNA expression of IL-17A and its receptor IL-17RA, but not IL-17RC, was markedly upregulated in the PVN of HF when compared with SHAM rats. Genetic knockdown of IL-17RA by bilateral PVN microinjections of an IL-17RA siRNA AAV virus attenuated mRNA expression of proinflammatory cytokines and chemokines, and ameliorated sympathetic activation and cardiac function in HF rats. These data indicate that elevated expression of IL-17A in the brain in HF contributes to the excessive central inflammatory state and cardiac dysfunction in HF. Interventions to suppress IL-17A/IL-17RA axis in the brain have the potential for treating HF.

## Introduction

Inflammation-driven sympathetic activation contributes largely to the cardiac dysfunction and remodeling of heart failure (HF). Our work over the past decade in animal studies has revealed that blood-borne proinflammatory cytokines can access the central nervous system (CNS), more prominently in the cardiovascular/autonomic centers of the brain such as the subfornical organ and hypothalamic paraventricular nucleus (PVN), leading to the neurohumoral activation (Yu et al., 2010; Wei et al., 2013) – a key hallmark and well-documented determinant of HF progression. The cytokine IL-17A is a key inflammatory mediator linking immune responses with tissue inflammation (Yu and Gaffen, 2008). We previously reported that peripherally administered IL-17A acts in the brain to amplify the expression of inflammatory cytokines and chemokines produced by astrocytes and microglia to promote neuroinflammation and sympathetic outflow, and IL-17A-elicited neuroinflammation plays an important role in the development of ANG-II induced hypertension (Cao et al., 2021(Cao et al., 2021). However, whether IL-17A contributes to the neuroinflammation and autonomic activation in the context of HF remains uninvestigated.

IL-17A (commonly known as IL-17) is the founding member of the IL-17 family, composed of 6 isoforms from IL-17A to 17F (Aggarwal and Gurney, 2002). IL-17A exists in the form of homodimer or heterodimer with its homolog IL-17F to activate a receptor complex constructed with IL-17 receptors A and C (IL-17RA and IL-17RC) (Toy et al., 2006; Wright et al., 2008). Both IL-17A and F are secreted mainly by a unique subset of activated CD4 + T helper 17 (Th17) cells. Although IL-17A was initially identified as a critical inflammatory mediator in the pathogenesis of several autoimmune diseases (Raychaudhuri, 2013; Luchtman et al., 2014), emerging evidence indicates that IL-17A also contributes to the pathophysiology of cardiovascular diseases, including cardiac ischemia/reperfusion injury, atherosclerosis, myocarditis, and dilated cardiomyopathy (Liao et al., 2012; Wu et al., 2014; Robert and Miossec, 2017), primarily by evoking substantial production of various inflammatory mediators.

Over the past decade, IL-17A has been associated with the disease process of HF in humans and animal models (Avalos et al., 2012; Chang et al., 2018). IL-17A levels are elevated in both injured hearts and circulation. Myocardial infraction (MI)-induced HF causes tissue injury and generates damage signals that trigger immune responses and infiltration of immune cells such as leukocytes, neutrophils, monocytes, and macrophages to the injured sites. In chronic HF myocardium, Th17 cells, the main IL-17A-producing cells, are essential for the development of immune reaction, and macrophages and cardiomyocytes are the major sources of inflammatory cytokines in response to IL-17A (Mora-Ruiz et al., 2019). It should be noted that elevated levels of cytokines in tissues and circulation can affect cardiac function farther afield than just locally in the peripheral organs. In this regard, IL-17A is of particular significance because of its distinctive ability to disrupt the integrity of the blood brain barrier (BBB) to reach the brain (Kebir et al., 2007; Setiadi et al., 2019). Work from our lab and others has shown that receptors for IL-17A are richly expressed in the brain, and more prominently distributed throughout the PVN (Cao et al., 2021), a key autonomic brain center where inflammation is known to drive sympathetic overactivity.

The present study sought to determine whether IL-17A and its receptors are upregulated in the brain in HF-induced by MI and, if so, whether increased IL-17A mediates the neuroinflammation and sympathetic excitation that contribute to the cardiac dysfunction in of this disease setting. We hypothesized that elevated IL-17A binding to IL-17RA in the brain induces the production of various inflammatory mediators that cause a prolonged inflammatory state. Central interventions suppressing the IL-17/IL-17RA axis ameliorate neuroinflammation and cardiac function in this model of HF. PVN was used as the primary CNS target for IL-17A action and the central site of intervention with IL-17RA siRNA.

## Materials and methods

### Animals

Adult male Sprague-Dawley rats (10 weeks) were purchased from Envigo/Harlan (Indianapolis, IN). Animals were housed in temperature- (23 ± 2°C) and humidity-controlled light-cycled rooms (12:12-h light-dark cycle). Standard rat chow and water were provided *ad libitum*. All experimental procedures in this study were carried out in accordance with the National Institutes of Health Guide for the Care and Use of Laboratory Animals and were approved by the Institutional Animal Care and Use Committee of the University of Iowa.

### Experimental protocols

#### Protocol I: Assessments of IL-17A levels and expression of its receptors in the brain in heart failure

Under ketamine plus xylazine (100 + 10 mg/kg) anesthesia, rats underwent left coronary artery ligation (CL) to induce HF (*n* = 16) or operation (SHAM, *n* = 11). Within 24 h of CL, and again 4 weeks after CL, cardiac injury and function were assessed by echocardiogram. Animals with a small ischemic zone (≤25%) or that died before the end of the experiments were excluded from studies (*n* = 4). At the termination of the protocols, SHAM (*n* = 7) and surviving HF rats (n = 8) were sacrificed to collect cerebrospinal fluid (CSF), brain, and blood for molecular measurements of levels of IL-17A or its receptors in the plasma and brain. The heart and lungs were also harvested and weighed to evaluate peripheral indicators of HF. Additionally, some SHAM (*n* = 4) and HF (*n* = 4) were transcardially perfused with 4% paraformaldehyde under deep anesthesia for immunofluorescent staining to evaluate IL-17RA expression in the PVN.

#### Protocol II: Validation of the efficacy of IL-17A siRNA AAV virus in paraventricular nucleus in normal rats

Rats anesthetized with ketamine plus xylazine (100 + 10 mg/kg) underwent bilateral PVN microinjections of an adeno-associated virus 9 (AAV9) vectors encoding either an IL-17RA siRNA (0.3 μl of 10^13^ GC/ml, *n* = 9) or a scrambled (Scr) siRNA (*n* = 9) with green fluorescent protein (GFP). Normal rats (*n* = 9) received no treatment served as controls. One week later, these rats were euthanized by decapitation to collect brains to verify the transfection potential and knockdown efficacy of AAV-IL-17RA siRNA.

#### Protocol III: Assessment of the effect of genetic knockdown of IL-17RA in the paraventricular nucleus by IL-17RA siRNA AAV virus on neuroinflammation and cardiac function in heart failure

Rats received bilateral PVN microinjections of an IL-17RA siRNA (0.3 μl 10^13^ GC/ml, *n* = 20) or a Scr siRNA (*n* = 19) AAV9 vector as described in protocol II. One week later, these animals underwent CL to induce HF or SHAM. Cardiac injury and function were assessed by an echocardiogram within 24 h of CL. Animals with a small ischemic zone (≤25%) or that died before the end of the experiments were excluded from studies (*n* = 7). The groups and final animal numbers used for data analysis were as follows: (1) HF + IL-17RA siRNA (*n* = 8); (2) HF + Scr siRNA (*n* = 8); (3). SHAM + IL-17RA siRNA (*n* = 8); and (4) SHAM + Scr siRNA (*n* = 8).

Four weeks after MI, an echocardiogram and hemodynamic measurement for assessments of cardiac function and sample collections for molecular studies were performed as described in Protocol I.

### Heart failure induction and echocardiography

Rats were anesthetized with a mix of ketamine and xylazine (90 mg/kg + 10 mg/kg, ip). After the chest was opened and the pericardium was removed, the left anterior descending coronary artery was ligated with a 6-0 silk suture to induce HF, whereas the same surgical procedures except for the ligation of coronary artery were performed to produce SHAM rats, as previously described (Yu et al., 2019b). Within 24 h and 4 weeks after coronary artery ligation, animals were anesthetized again with ketamine (60 mg/kg ip) and echocardiography was carried out to assess cardiac injury and function, including ischemic zone as a percent of left ventricular (LV) circumference (% IZ), LV ejection fraction (LVEF) and LV end-diastolic volume (LVEDV), as previously described (Yu et al., 2017, 2019b).

### Cardiac hemodynamic and anatomical assessments

At the end of the study protocols, animals were anesthetized with urethane (1.5 g/kg ip). A Millar catheter was inserted into the right carotid artery and then advanced into the LV. Blood pressure, heart rate, LV peak systolic pressure, LV end-diastolic pressure, and maximum rate of rise of LV pressure (LV dP/dt_max_) were measured as previously described (Yu et al., 2017). Immediately after hemodynamic measurements, animals were sacrificed and heart and lungs were collected and weighed. The ratios of LV weight-to-body weight (BW), right ventricular (RV) weight-to-BW, and wet lung weight-to-BW were determined to indicate cardiac remodeling and pulmonary congestion, the important characteristics of systolic HF.

### Surgical preparation for bilateral PVN microinjection of IL-17 siRNA AAV vector

Bilateral PVN microinjections of an IL-17RA siRNA or a Scr siRNA (Cat. No. iAAV06222909 and iAAV01509, respectively, Applied Biological Materials Inc., Richmond, BC, Canada) AAV9 vector encoding GFP was carried out as previously described (Cao et al., 2021). Briefly, animals were placed in a stereotaxic instrument and the skull was exposed through an incision on the midline of the scalp. After two small holes were drilled at 1.8 mm posterior to bregma and 0.4 mm from midline, a 29-gauge guide cannula was inserted to a position 5.8 mm ventral to dura and a 35-gauge (128 μm outer diameter, and 51.2 μm inner diameter) injection cannula connected to a 1.0 μl micro-syringe was advanced into the PVN through the guide cannula. The tip of the injection cannula was adjusted to a length extending 2 mm beyond the tip of the guide cannula. IL-17RA siRNA and Scr siRNA AAV vectors were bilaterally microinjected into the PVN over 30 s and the injection cannula was left at the injection site for additional 5 min before removing from the PVN. The microinjection sites in the PVN were verified by GFP expression.

### Real-time polymerase chain reaction

Brains were quickly removed and frozen in liquid nitrogen. The PVN regions, including small amounts of surrounding tissues, were punched from the frozen brain using a 15-gauge needle stub (inner diameter, 1.5 mm). Total RNA was extracted from PVN punches with the RNeasy^®^ Plus Mini Kit (QIAGEN, Germantown, MD). mRNA expression for inflammatory markers tumor necrosis factor (TNF)-α, interleukin (IL)-1β, IL-6, monocyte chemoattractant protein (MCP)-1, macrophage inflammatory protein (MIP)-1α and chemokine stromal cell-derived factor (SDF)-1, and mRNA expression for neuronal activity marker c-Fos in the PVN were analyzed with SYBR Green real-time PCR after reverse transcription of total RNA as previously described (Yu et al., 2019b). Primers for rat IL-17A, IL-17RA and IL-17RC were purchased from Bio-Rad Laboratories (Hercules, CA). Sequences for the rest of primer pair are presented in Table 1. Real-time PCR was performed using the QuantStudio 3 Real-Time PCR System (Applied Biosystems, Carlsbad, CA). mRNA data were corrected by β-actin and expressed as fold changes relative to SHAM, control, or SHAM + Scr siRNA groups.

**TABLE 1:** Sequences for primers.

*Gene*	*Primers*	*Sequences*
TNF-α	Forward primer: Reverse primer:	5′-CCTTATCTACTCCCAGGTTCTC-3′ 5′-TTTCTCCTGGTATGAATGGC-3′
IL-1β	Forward primer: Reverse primer:	5′-CGACAGAATCTAGTTGTCC-3′ 5′-TCATAAACACTCTCATCCACAC-3′
IL-6	Forward primer: Reverse primer:	5′-TCCTACCCCAACTTCCAATGCTC-3′ 5′-TTGGATGGTCTTGGTCCTTAGCC-3′
MCP-1	Forward primer: Reverse primer:	5′-CAGATCTCTCTTCCTCCACCACTAT-3′ 5′-CAGGCAGCAACTGTGAACAAC-3′
MIP-1α	Forward primer: Reverse primer:	5′-GCGCTCTGGAACGAAGTCT-3′ 5′-GAATTTGCCGTCCATAGGAG-3′
SDF-1	Forward primer: Reverse primer:	5′-TGGTTCAGAGGATCGTCCAAA-3′ 5′-CAGGAGCCCATGTTCTTCCTT-3′
c-Fos	Forward primer: Reverse primer:	5′-GTCAACACACAGGACTTTTGCG-3′ 5′-CGTGGGGATAAAGTTGGCACT-3′
β-actin	Forward primer: Reverse primer:	5′-CCGCGAGTACAACCTTCT-3′ 5′-CGTCATCCATGGCGAACT-3′

### Immunofluorescent studies

To determine IL-17RA expression in the PVN, rats were transcardially perfused with 4% paraformaldehyde. Brains were removed, post-fixed in 4% paraformaldehyde for 24 h at 4°C and then cryoprotected in 30% sucrose for 48 h at 4°C. Brains were sectioned into 20 μm coronal sections using a cryostat. The sections were incubated with a rabbit anti-rat primary antibody to IL-17RA (ab180904, 1:50, Abcam, Cambridge, MA) and a mouse anti-rat primary antibody to NeuN (MAB377, 1:200, Millipore, Billerica, MA), followed by secondary antibodies Alexa fluor 488 goat anti-rabbit IgG (ab150077, 1:200, Abcam, Cambridge, MA) and Alexa fluor 568 goat anti-mouse IgG (ab 175702, 1:200, Abcam), respectively. To verify the transfection potential of the siRNAs by visualization of GFP in the PVN, rats were sacrificed without perfusion and brains were removed quickly. Brains were immediately frozen in liquid nitrogen and then sectioned into 20 μm coronal sections using a cryostat. Immunofluorescence for IL-17RA and NeuN and GFP were visualized by a confocal laser-scanning microscope (Zeiss LSM 710, Carl Zeiss, Inc., Oberkochen, Germany).

### Biochemical assays

CSF was collected from the cisterna magna (Pegg et al., 2010). Under anesthesia with urethane (1.5 g/kg ip), rats were placed in a stereotaxic frame and secured with an ear bar. The rat skull was positioned downward at a 45° angle. One midline incision was made to expose the atlanto-occipital membrane. CSF was withdrawn from the cisterna magna using a 26G needle connected with a 1-ml syringe. The color of the CSF samples was closely observed to avoid any possible blood contamination.

IL-17A levels in CSF and plasma and norepinephrine (NE) levels in plasma were determined by a rat IL-17A ELISA kit (ab119536, Abcam) and a rat NE ELISA kit (BA 10-5200, LDN, Germany), according to the manufacturers’ instructions.

### Statistical analysis

All data are presented as mean ± SEM. Analysis of the molecular, biochemical, and histologic measurements were performed blindly. Kolmogorov-Smirnov test and Levene’s test were conducted to verify normal distributions and equal variances, respectively. Statistical analyses were performed using Student’s *t*-test, one-way or two-way ANOVA followed by Tukey’s multiple comparison tests. linear regression analysis was applied to assess associations between plasma IL-17A levels and CSF IL-17A levels. *p* < 0.05 was considered statistically significant.

## Results

### Echocardiographic and anatomical assessments of heart failure

Echocardiographic assessment of LV function in HF was performed within 24 h and 4 weeks after CL (Table 2). Within 24 h of CL, large ischemic zone (%IZ >25%), reduced LVEF, and increased LVEDV were found in ischemia-induced HF compared with SHAM rats. Four weeks after CL, although there was no significant change in ischemic zone, HF rats experienced an additional reduction in LVEF and an increase in LVEDV when comparing with the measurements within 24 h after CL, consistent with progression of HF.

**TABLE 2:** Echocardiographic and anatomical measurements.

Variables	SHAM	HF
**Echocardiographic variables at 24 h**		
%IZ	—	42 ± 3*
LVEF	0.85 ± 0.03	0.39 ± 0.02*
LVEDV (ml)	0.37 ± 0.02	0.69 ± 0.03*
**Echocardiographic variables at week 4**		
%IZ	—	44 ± 2*
LVEF	0.86 ± 0.03	0.27 ± 0.03*§
LVEDV (ml)	0.43 ± 0.03	1.11 ± 0.06*§
**Anatomical variables at week 4**		
BW (g)	418 ± 6	405 ± 5
LV/BW (mg/g)	2.18 ± 0.05	2.26 ± 0.06
RV/BW (mg/g)	0.46 ± 0.02	0.84 ± 0.06*
Lung/BW (mg/g)	3.00 ± 0.07	8.12 ± 0.99*

%IZ: ischemic zone as a percent of left ventricular (LV) circumference; LVEF: LV ejection fraction; LVEDV: LV end-diastolic volume. BW: body weight; LV: left ventricular; RV: right ventricular.

Values are expressed as mean ± SEM (*n* = 7-8 for each group).

*p < 0.05 versus SHAM at same week; ^§^*p* < 0.05 HF at 4 weeks versus 24 h.

At the end of the experiment, ratios of LV-to-BW, RV-to-BW, and wet lung-to-BW were measured in HF and SHAM rats (Table 2). These anatomical measurements revealed similar BW and the ratio of LV-to-BW in SHAM and HF animals at 4 weeks after CL. However, HF rats displayed RV hypertrophy as evidenced by an increased ratio of RV-to-BW and pulmonary congestion as indicated by a greater ratio of lung-to-BW.

### IL-17A levels in plasma and cerebrospinal fluid in heart failure

**FIGURE 1:**
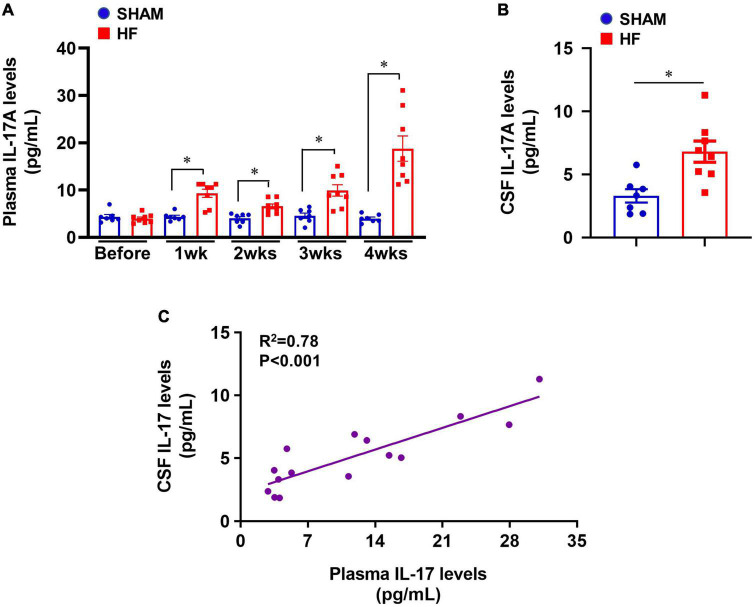
**(A)** Plasma IL-17A levels in SHAM and HF rats at 1-4 weeks after CL. **(B)** CSF IL-17A levels in SHAM and HF rats 4 weeks after CL. **(C)** Correlation between plasma IL-17A levels and CSF IL-17A levels in SHAM and HF rats 4 weeks after CL. CSF: cerebrospinal fluid. Data are mean ± SEM (*n* = 7-8 for each group); *p < 0.05.

Compared with SHAM rats, plasma levels of IL-17A were significantly increased in HF rats beginning within 1 week after CL (Figure 1A) and were sustained at a higher level than baseline level for the remainder of next 3 weeks. The greatest increases in plasma levels of IL-17A were observed 4 weeks after CL. Meanwhile, CSF levels of IL-17A were also increased in HF rats when compared with SHAM rats 4 weeks after CL (Figure 1B). Linear regression analysis indicated that IL-17A levels in CSF were positively correlated with increased IL-17A levels in plasma (Figure 1C).

### Expression of IL-17A and its receptors in the paraventricular nucleus in heart failure

**FIGURE 2:**
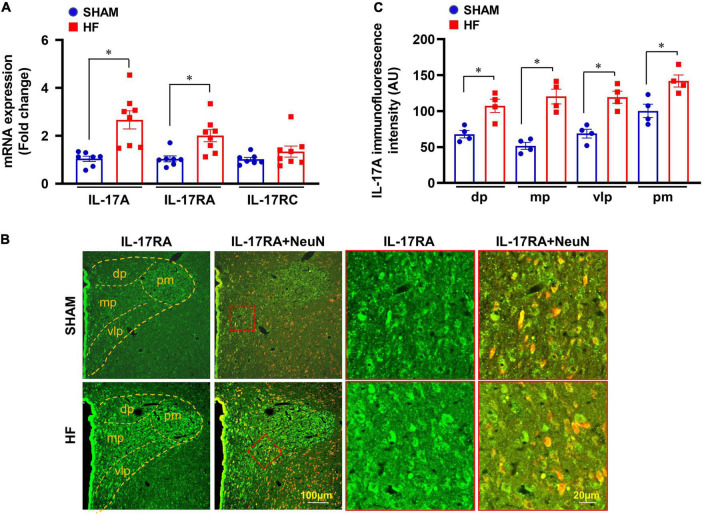
**(A)** mRNA expression of IL-17A and its receptors IL-17RA and IL-17RC in the PVN of SHAM and HF rats 4 weeks after CL. **(B)** Representative confocal images showing expression of IL-17RA (green) and NeuN (a neuronal marker, red) and merged images (yellow) in the PVN of SHAM and HF rats 4 weeks after CL. **(C)** IL-17RA immunofluorescence intensity in 4 subnuclear regions of the PVN including dorsal parvocellular (dp), medial parvocellular (mp), ventrolateral parvocellular (vlp), and posterior magnocellular (pm), in SHAM and HF rats 4 weeks after CL. Values are expressed as means ± SEM (*n* = 4-8 for each group); *p < 0.05.

Four weeks after CL, mRNA expression of IL-17A and its receptor IL-17RA was markedly upregulated in the PVN of HF rats compared with SHAM rats (Figure 2A). Although IL-17A receptor IL-17RC mRNA in the PVN showed a trend toward an increase, there was no significant difference between HF and SHAM animals.

Confocal immunofluorescent images demonstrated that IL-17RA was substantially expressed in neuronal and non-neuronal elements of PVN in both SHAM and HF rats (Figure 2B). However, the fluorescent intensity for IL-17RA in the dorsal parvocellular, medial parvocellular, ventrolateral parvocellular, and posterior magnocellular regions, the four commonly recognized subdivisions of the PVN, was significantly higher in HF rats compared with SHAM rats (Figure 2C).

### Validation of IL-17RA knockdown by its siRNA in the paraventricular nucleus

**FIGURE 3:**
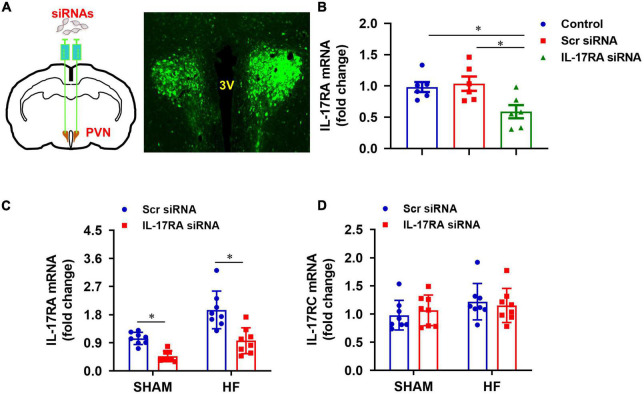
**(A)** Schematic diagram showing bilateral PVN microinjection sites and GFP expression in the PVN, and **(B)** effect of bilateral PVN microinjection of IL-17RA siRNA on mRNA expression of IL-17A receptor IL-17RA in normal rats 1 week after bilateral PVN microinjections of IL-17RA siRNA AAV virus. **(C,D)** Effect of bilateral PVN microinjection of IL-17RA siRNA on mRNA expression of IL-17A receptors IL-17RA and IL-17RC in the PVN of SHAM and HF rats 4 weeks after CL. 3V: third cerebroventricle; Scr: scrambled siRNA. Values are mean ± SEM (*n* = 6-8 for each group) and are expressed as a fold change compared to control or SHAM + Scr siRNA; *p < 0.05.

To determine a role of IL-17A in the brain, we genetically knocked down its receptor, IL-17RA, in the PVN with an IL-17RA siRNA AAV viral vector tagged with GFP. The transfection potential and efficacy were verified in normal rats prior to the experimental protocols in HF rats. As shown in Figure 3A, GFP fluorescence was found to be distributed diffusely throughout the PVN of rats 1 week after bilateral PVN microinjections of IL-17RA siRNA, confirming the accurate microinjection sites and effective viral transfection in the PVN neurons. Compared with untreated normal rats, rats treated with IL-17RA siRNA had significantly reduced IL-17RA mRNA expression (Figure 3B) in the PVN, but not the rats treated with Scr siRNA.

Genetic knockdown of IL-17RA in the PVN by its siRNA was tested in HF and SHAM rats. Four weeks after CL, Scr siRNA-treated HF rats had significantly higher mRNA levels of IL-17RA in the PVN, compared with Scr siRNA-treated SHAM rats (Figure 3C). IL-17RA mRNA levels in the PVN in both SHAM and HF animals were significantly reduced by bilateral PVN microinjections of IL-17RA siRNA. The IL-17RC mRNA levels of the PVN in both SHAM and HF animals were not affected by bilateral PVN microinjections of IL-17RA siRNA, indicating the specificity of IL-17RA siRNA to reduce the expression of IL-17RA in the PVN (Figure 3D).

### Effect of paraventricular nucleus IL-17RA knockdown on the expression of inflammatory mediators

**FIGURE 4:**
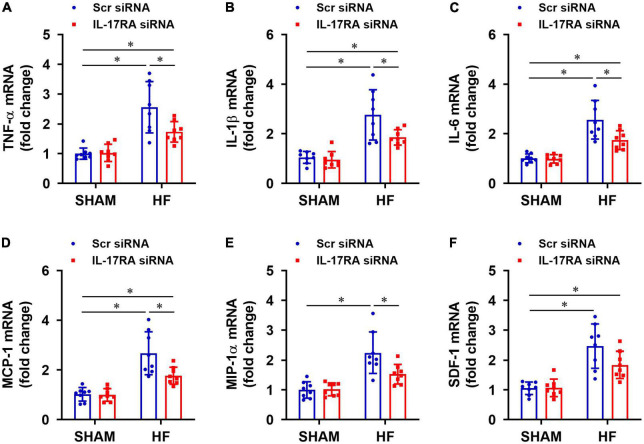
Effect of bilateral PVN microinjection of IL-17RA siRNA on mRNA expression of proinflammatory cytokine tumor necrosis factor (TNF)-α **(A)**, interleukin (IL)-1β **(B)** and IL-6 **(C)**, and on mRNA expression of chemokines monocyte chemoattractant protein-1 (MCP-1, **D**), macrophage inflammatory protein-1α (MIP-1α, **E**) and chemokine stromal cell-derived factor-1 (SDF-1, **F**) in the PVN of SHAM and HF rats 4 weeks after CL. Scr: scrambled siRNA. Values are mean ± SEM (*n* = 8 for each group) and are expressed as a fold change compared to SHAM + Scr siRNA; *p < 0.05.

Rats assigned to the HF treatment groups were well-matched with regard to size of the ischemic zone and LVEF. Four weeks after CL, HF + Scr siRNA rats exhibited markedly increased mRNA expression of inflammatory cytokines TNF-α (Figure 4A), IL-1β (Figure 4B), IL-6 (Figure 4C), and chemokines MCP-1 (Figure 4D), MIP-1α (Figure 4E), SDF-1 (Figure 4F) in the PVN, compared with SHAM + Scr siRNA rats. Treatment with bilateral PVN microinjections of IL-17RA siRNA significantly reduced mRNA expression of these measured inflammatory cytokines and chemokines in the PVN in HF rats, with the exception of SDF-1 that was not statistically different. Bilateral PVN microinjections of IL-17RA siRNA did not alter expression of these inflammatory mediators in SHAM rats.

### Effect of paraventricular nucleus IL-17RA knockdown on sympatho-excitatory mediators

**FIGURE 5:**
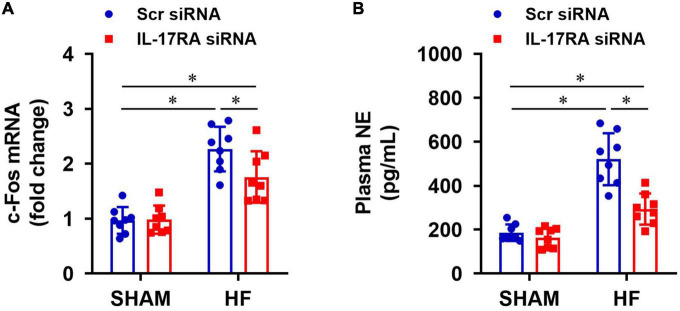
Effect of bilateral PVN microinjection of IL-17RA siRNA on mRNA expression of c-Fos (a marker of neuronal activity, **A**) in the PVN and on levels of plasma norepinephrine (NE, a marker of sympathetic nerve activity, **B**) in SHAM and HF rats 4 weeks after CL. Values are mean ± SEM (*n* = 8 for each group); *p < 0.05.

Compared with SHAM + Scr siRNA rats, HF + Scr siRNA rats had higher mRNA expression of c-Fos (a recognized indicator of neuronal activation) in the PVN (Figure 5A) and elevated levels of NE (an important marker of global sympathetic activity) in plasma (Figure 5B). c-Fos expression in the PVN and NE levels in the plasma were significantly decreased in HF rats treated with IL-17RA siRNA, compared with HF rats treated with Scr siRNA. There were no differences in mRNA expression of c-Fos in the PVN and levels of NE in plasma in the SHAM rats treated with IL-17RA siRNA.

### Effect of paraventricular nucleus IL-17RA knockdown on cardiac function and hemodynamics

**FIGURE 6:**
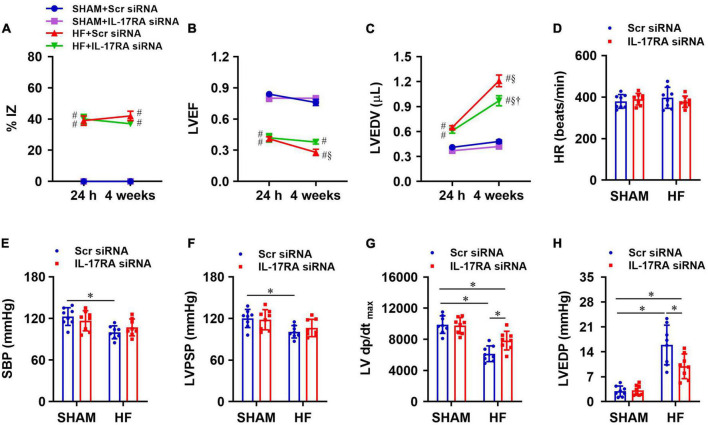
Quantitative comparison of echocardiographic variables including ischemic zone as a percent of left ventricular (LV) circumference (%IZ, **A**), LV ejection fraction (LVEF, **B**) and LV end-diastolic volume (LVEDV, **C**) 24 h and 4 weeks after coronary artery ligation. **(D–H)**: quantitative comparison of LV hemodynamic variables including heart rate (HR, **D**), systolic blood pressure (SBP, **E**), LV peak systolic pressure (LVPSP, **F**), maximum rate of rise of LV pressure (LV dP/dt_max_, **G**) and LV end-diastolic pressure (LVEDP, **H**) from each group 4 weeks after coronary artery ligation. Values are mean ± SEM (*n* = 8 for each group); # *p* < 0.05 vs. SHAM + Scr siRNA; §*p* < 0.05 vs. same group at 24 h; †*p* < 0.05, HF + IL-17RA siRNA vs. HF + Scr siRNA; *p < 0.05.

Within 24 h of CL, echocardiographic assessments revealed that the infarct size (%IZ, Figure 6A) and the degree of cardiac dysfunction as indicated by reduced LVEF (Figure 6B) and increased LVEDV (Figure 6C) was not significantly different between HF + Scr siRNA and HF + IL-17RA siRNA groups. Four weeks after CL, infarct size (%IZ) was unaltered in both HF + Scr siRNA rats and HF + IL-17RA siRNA rats. However, cardiac function in HF + Scr siRNA rats deteriorated with further decreased LVEF and increased LVEDV. HF rats treated with bilateral PVN microinjections of IL-17RA siRNA did not display further reduction of LVEF or an increment of LVEDV as seen in HF rats treated with control siRNA, when compared with these variables measured within 24 h after CL. Echocardiographic parameters of cardiac function were similar between SHAM + Scr siRNA and SHAM + IL-17RA siRNA.

Four weeks after CL, heart rate was similar in HF + Scr siRNA rats and SHAM + Scr siRNA rats (Figure 6D). SBP (Figure 6E), LVPSP (Figure 6F), and LV dP/dt_max_ (Figure 6G) were lower, and LVEDP (Figure 6H) was higher in HF rats treated with Scr siRNA vs. SHAM rats treated with Scr siRNA. HF rats treated with IL-17RA siRNA had higher LV dP/dt_max_ and lower LVEDP than HF rats treated with Scr siRNA, but these measurements were still significantly different than those of SHAM rats. Treatment with PVN IL-17RA siRNA in HF did not change SBP and LVPSP. There were no significant differences in hemodynamic parameters between SHAM groups.

### Effect of PVN IL-17RA knockdown on anatomic indicators

**FIGURE 7:**
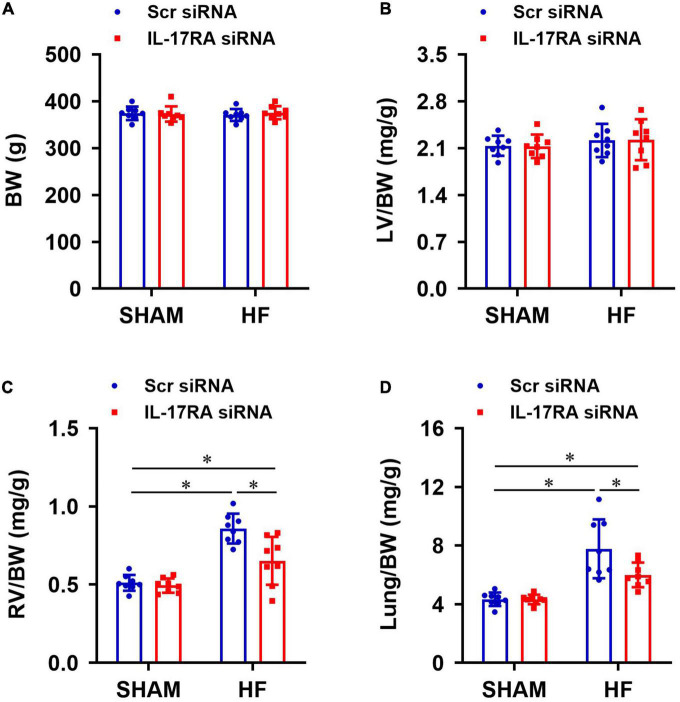
Anatomic measurements including body weight (BW, **A**), the ratios of left ventricular weight-to-BW (LV/BW, **B**), right ventricular weight-to-BW (RV/BW, **C**), and wet lung weight-to-BW (Lung/BW, **D**) from each group 4 weeks after coronary artery ligation. Values are mean ± SEM (*n* = 8 for each group); *p < 0.05.

Anatomical assessments following euthanasia indicated that there were no significant differences in BW (Figure 7A) or the ratios of LV-to-BW (Figure 7B) across all four experimental groups. The ratios of RV-to-BW (Figure 7C) and lung-to-BW (Figure 7D) were substantially higher in HF + Scr siRNA than SHAM + Scr siRNA rats. Treatment with bilateral PVN IL-17RA siRNA in HF rats reduced the ratios of RV-to-BW and lung-to-BW when compared with HF rats treated with Sci siRNA. These measurements did not alter in SHAM rats treated with IL-17RA siRNA in the PVN.

## Discussion

Sympathetic nervous system activation plays an important role in cardiovascular dysfunction and is associated with increased morbidity and mortality (Malpas, 2010). Proinflammatory cytokine-induced sympathetic overactivity and neurohumoral activation contribute significantly to the progression of HF (Wei et al., 2012; Yu et al., 2017, 2019a; Haspula and Clark, 2018). In this work, we investigated the role of IL-17A in mediating neuroinflammation and neurohumoral activation in the brain to identify the central inflammatory mechanisms underlying the development of HF. The main findings of this study are: (1) MI induces elevated protein levels of IL-17A in plasma and CSF, along with increased gene expression of IL-17A and its receptor, IL-17RA, in the PVN. The increased IL-17A in the brain in the context of HF is associated with its concentration in the plasma; and (2) genetic knockdown of IL-17RA in the PVN attenuates the increases in gene expression of inflammatory cytokines and chemokines, and ameliorates sympathetic-excitatory mediators and peripheral manifestations of the heart in HF. Our study suggests that the upregulated IL-17A in the periphery of MI-induced HF gains access to the brain where it triggers the production of a broad spectrum of proinflammatory cytokines and chemokines. These inflammatory mediators produced in the brain activate autonomic and neuroendocrine neurons, particularly in the PVN, to advance sympathetic and hormonal activation, thereby leading to the deterioration of cardiac function in HF.

Augmented levels of IL-17A have been reported in peripheral tissues and the central nervous system in some autoimmune and neurodegenerative disorders, such as psoriasis, rheumatoid arthritis and multiple sclerosis (Luchtman et al., 2014; Beringer et al., 2016; Blauvelt and Chiricozzi, 2018). In the present study, we found that levels of IL-17A in plasma were also significantly increased during the progression of HF, initiating within 1 week with a peak level measured at 4 weeks after myocardial infarction. These findings were consistent with previous reports in animal models and humans showing increased IL-17A levels in circulation and the infarcted heart (Bochaton et al., 2017; Chang et al., 2018). It should be noted that although increased levels of IL-17A in plasma and the heart are initially associated with an increased number of IL-17A producing cells in the injured tissues of the failing heart (Chang et al., 2018), as HF progresses, immune cells in the blood and gut also produce IL-17A. Changes in composition and diversity of gut microbiota in HF has been reported to drive the production of IL-17A (Ahmad et al., 2008; Calcinotto et al., 2018).

Importantly, our data extend previous findings by displaying that the levels of IL-17A in the brain CSF and PVN were also significantly elevated after myocardial infarction, which correlate well with its levels measured in the plasma. The elevated levels of IL-17A in the circulation may contribute partly to the high protein levels of IL-17A in the brain. Due to the protection by the BBB, however, how IL-17A, the large molecule in the periphery, reaches the brain remains unclear. IL-17A levels in CSF have been reported to be associated with breakdown of the BBB in animal models of relapsing-remitting multiple sclerosis (Setiadi et al., 2019). Many other studies also demonstrate that the higher levels of IL-17 can impair the BBB to reach the brain. We acknowledge that the circulating IL-17A in this model of HF might enter the brain through similar mechanisms by disrupting the integrity of the BBB. Several studies have demonstrated that IL-17A can access the brain by downregulating integral plasma-membrane proteins at tight junctions on endothelial cells of the BBB (Kebir et al., 2007; Huppert et al., 2010; Setiadi et al., 2019). It is also possible that IL-17A accesses the brain via circumventricular organs (CVOs), the intrinsic brain structures that lack BBB and allow rapid neurohumoral exchange between the CNS and circulation (Johnson and Gross, 1993; Ferguson and Bains, 1996). Additionally, BBB dysfunction is found in some pathological conditions such as heart failure and hypertension (Liu et al., 2013; Setiadi et al., 2018), so that IL-17A in the periphery may more readily penetrate the BBB to reach the brain. Those considerations notwithstanding, we show here, using siRNA knockdown targeted to PVN, that local expression of IL-17A modulated CNS neuroinflammation and sympathetic activation in HF.

Although both IL-17RA and IL-17RC are expressed in the CNS, it appears that IL-17RA might play a dominant role rather than IL-17RC in mediating the action of IL-17A to promote inflammatory response in the brain. In our study, IL-17RA was substantially expressed throughout the PVN and upregulated in HF rats after MI, as assessed by either real-time PCR or immunofluorescent staining. In spite of the fact that the cellular types of the PVN were not identified, neurons as well as microglia and astrocytes would be the cells expressing IL-17RA. It is well-documented that glial cells are the main immune cells in the brain that produce the inflammatory mediators needed to promote neuroinflammatory conditions (Carson et al., 2006; Kwon and Koh, 2020). Therefore, the elevated expression of inflammatory cytokines and chemokines in the brain in HF is likely caused by the activation of microglia and astrocytes when upregulated IL-17A binds to its receptors in these brain-resident immune cells.

To determine the contribution of elevated IL-17A to neuroinflammation in the brain in HF, we performed genetic knockdown of IL-17RA by bilateral PVN microinjections of an IL-17RA siRNA AAV9 virus. Since AAV9 was reported to target both neurons and glial cells (Aschauer et al., 2013; Dashkoff et al., 2016), it is believed that the expression of IL-17RA in all these brain-resident cells in PVN would be reduced by IL-17RA siRNA. The efficacy of IL-17RA siRNA to knock down IL-17RA in PVN had also been tested and verified in our previous study in normal and ANG II-induced hypertensive rats (Cao et al., 2021). Our data indicate that reduction of IL-17A expression reduced the elevated levels of inflammatory cytokines and chemokines in the PVN in HF, but had no effects on these inflammatory mediators in SHAM rats, suggesting that IL-17A is a major player in mediating neuroinflammatory condition of the brain in the context of HF. It is worth noting that the levels of most proinflammatory mediators in the PVN in HF animals treated with IL-17RA siRNA still remain higher than those in SHAM animals, implying that other mechanisms such as the brain renin-angiotensin system may also contribute to the neuroinflammatory response in the brain in HF (Yu et al., 2018; Diaz et al., 2020). Knockdown of IL-17RA in the PVN of HF rats also reduced sympathetic excitation, as evidenced by decreased plasma levels of NE (a recognized indicator for global sympathetic nerve activity) and the expression of immediate early gene c-Fos in the PVN (a molecular marker for neuronal activity). PVN is a major pre-sympathetic region of the brain and has been implicated in the altered regulation of sympathetic nerve activity and extracellular fluid volume in HF (Li and Patel, 2003; Ramchandra et al., 2013). Taken together, these data suggest that IL-17A-induced neuroinflammation in cardiovascular/autonomic regions of the brain contribute to sympathetic and humoral activation in HF. IL-17A-induced global sympathetic activation may play a critical role in IL-17A-mediated effects on cardiac dysfunction in HF.

Reduction of IL-17A expression markedly ameliorated cardiac dysfunction and remodeling in HF. Bilateral microinjection of IL-17RA in the PVN prevented further deterioration in LVEF and LVEDV, and improved cardiac hemodynamics and contractility as indicated by reduced LVEDP and increased LV dP/dt_max_, respectively. The reduced ratios of the lung-to-body weight and the heart-to-body weight implicated improvements in pulmonary congestion and cardiac remodeling, which were consistent with cardiac dynamic changes. These improvements in peripheral manifestations in HF likely reflect the reduction in preload and afterload of the heart when sympathetically-mediated renal sodium retention and vasoconstriction were alleviated by suppressing the IL-17A/IL-17RA signaling in the brain.

*Limitations of the study:* While IL-17A in both periphery and the brain was shown to be upregulated in this HF model induced by MI, the mechanism underlying the involvement of immune processes and immune cells in producing IL-17A in infarcted tissues was not elucidated. However, this may be a potential direction to investigate the inflammatory mechanisms driven by IL-17A in HF. Additionally, although our data suggests that the upregulated expression of IL-17A in the brain in HF is associated with elevated levels of IL-17A in the circulation, how blood-borne IL-17A penetrates the BBB to reach the brain was not investigated. It needs to be mentioned that since mRNA levels of inflammatory cytokines and chemokines in the PVN have paralleled the protein levels when both were assessed in our previous study in this chronic HF model (Yu et al., 2017), only mRNA levels of these inflammatory mediators were measured in this work. Finally, this study was carried out in male animals only. Further studies in female animals are needed to determine whether sex differences exist regarding the role of the IL-17A/IL-17RA axis in the brain in promoting neuroinflammation, sympathetic excitation, and cardiac dysfunction in HF.

### Perspectives

The present study provides direct evidence for IL-17A as a key inflammatory regulator in enhancing central inflammatory states and driving sympathetic and hormonal activation in HF. The IL-17A/IL-17RA axis in the brain may represent a novel therapeutic target for cardiovascular diseases such as HF and hypertension. Given the largely successful amelioration of autoimmunity in psoriasis and ankylosing spondylitis by treatments with secukinumab and ixekizumab (monoclonal antibody inhibitors of IL-17A) and brodalumab (monoclonal antibody inhibitor for IL-17RA (Baeten et al., 2015; Bauer et al., 2015), the identification of IL-17A as a target for the treatment of HF would have significant potential for rapid translation in the clinical setting. Furthermore, this study will open a new avenue for the investigation of inflammatory mechanisms, not only in cardiovascular diseases such as HF and hypertension, but also in autoimmune diseases (e.g., multiple sclerosis) and neurodegenerative diseases (e.g., Parkinson’s disease), in which IL-17A is a critical contributor to the pathogenesis of the disease.

## Data availability statement

The original contributions presented in this study are included in the article/supplementary material, further inquiries can be directed to the corresponding author.

## Ethics statement

The animal study was reviewed and approved by Institutional Animal Care and Use Committee of the University of Iowa.

## Author contributions

S-GW and YY conceived and designed the research, interpreted the results of experiments, drafted the manuscript, and edited the manuscript. YY performed the experiments and prepared the figures. YY and RW analyzed the data. YY, RW, and S-GW revised and approved the final version of the manuscript. All authors contributed to the article and approved the submitted version.
